# Dental Literature

**Published:** 1870-10

**Authors:** 


### DENTAL LITERATURE.

In a profession so comparatively young as Dentistry, it is not unnatural that contributors to Dental literature manifest a disposition to diffusiveness and verbosity. The child with the new tin trumpet makes the most noise.

It should occur to occasional writers that there is no special reason why the treatment of a subject in Dentistry must be loaded down with verbiage, permeated with egotism, embittered with personality, ornamented with classic allusions and poetical quotations, and in short so disguised by clumsy attempts at explanation, that the idea loses identity, and the reader turns away with a sense of weariness and utter unprofitableness.

This will strike the readers of Dental periodicals. The standard works are few as compared with any department of medicine, yet they should be the guide and standard of composition. Anything that is known of famous writers in the world of science shows that in the process of composition they are laborious and conscientious in a high degree. The most distinguished writers use no more language than is absolutely necessary to plainly and clearly express an idea, while in description they are scrupulously exact, terse and brief.

To sit down and dash off the details of a case, or the report of a discussion before an association, and send it to a journal for publication before the ink is fairly dry upon the paper, is in no sense of the term writing; nor should such hasty contributors feel disappointed or chagrined upon either not seeing their undigested stuff in print, or finding it "razeed."

What is most needed is careful and deliberate writing, and this involves the frequently disregarded duty of legible penmanship and good spelling. We are frequently tempted to print *verbatim, et literatim, et spellatim*, contributions to this journal, as an example and a warning to evil-doers, and if it were not for the overruling desire to promote the knowledge indispensable to Dental science, the experiment would have been tried long ago.

In conclusion we have to request attention to the hints thrown out, feeling well assured that every Dentist with taste and judgment will recognize their justness and second us in the effort to produce a creditable Dental literature.

